# The genetic epidemiology of obsessive-compulsive disorder: a systematic review and meta-analysis

**DOI:** 10.1038/s41398-023-02433-2

**Published:** 2023-06-28

**Authors:** Thiago Blanco-Vieira, Joaquim Radua, Lívia Marcelino, Michael Bloch, David Mataix-Cols, Maria Conceição do Rosário

**Affiliations:** 1 Child and Adolescent Psychiatry Unit (UPIA), Department of Psychiatry, Federal University of São Paulo (UNIFESP), São Paulo, Brazil; 2 Institut d’Investigacions Biomediques August Pi i Sunyer, CIBERSAM, Instituto de Salud Carlos III, University of Barcelona, Barcelona, Spain; 3 Yale Child Study Center, Yale University, School of Medicine, New Heaven, USA; 4 Department of Clinical Neuroscience, Centre for Psychiatric Research, Karolinska Institutet, Stockholm, Sweden

**Keywords:** Psychiatric disorders, Clinical genetics

## Abstract

The first systematic review and meta-analysis of obsessive-compulsive disorder (OCD) genetic epidemiology was published approximately 20 years ago. Considering the relevance of all the studies published since 2001, the current study aimed to update the state-of-art knowledge on the field. All published data concerning the genetic epidemiology of OCD from the CENTRAL, MEDLINE, EMBASE, BVS, and OpenGrey databases were searched by two independent researchers until September 30, 2021. To be included, the articles had to fulfill the following criteria: OCD diagnosis provided by standardized and validated instruments; or medical records; inclusion of a control group for comparison and case-control, cohort or twin study designs. The analysis units were the first-degree relatives (FDRs) of OCD or control probands and the co-twins in twin pairs. The outcomes of interest were the familial recurrence rates of OCD and the correlations of OCS in monozygotic compared with dizygotic twins. Nineteen family, twenty-nine twin, and six population-based studies were included. The main findings were that OCD is a prevalent and highly familial disorder, especially among the relatives of children and adolescent probands, that OCD has a phenotypic heritability of around 50%; and that the higher OCS correlations between MZ twins were mainly due to additive genetic or to non-shared environmental components.

## Introduction

Obsessive-compulsive disorder (OCD) is a prevalent and highly heterogeneous disorder of unknown etiology. Similar to other psychiatric disorders, OCD probably originates from a complex interaction of genetic and environmental risk factors [1–3].

Since the beginning of the twentieth century, family studies have consistently reported that OCD is a familial disorder. However, the sample size, and methodological rigor of these studies have been mixed [4, 5]. Consequently, these studies have limited external validity [6]. More recently, population-based studies have significantly increased the sample sizes to several thousand probands and relatives but are limited by less precise diagnostic procedures [7–12].

Since OCD is a heterogeneous disorder, it may be possible that certain OCD phenotypes (i.e., early onset or tic-related OCD) are more familial/heritable than others [13–18]. However, not all studies had sufficient statistical power to confirm this familial pattern.

The first systematic review and meta-analysis of OCD genetic epidemiology were published approximately 20 years ago [19]. This study reported that FDRs of OCD probands had a four-fold higher risk for OCD than the FDRs of non-affected control probands. Since then, more than 20 relevant and high-quality original family studies [5, 7–18, 20–28] including two re-analyses of previous data have been reported [4, 29] and six population-based studies [7–12] have been published. And more than 25 high-quality twin studies [7, 30–56] have also been published.

Considering the relevance of all the studies published since 2001, the current study aimed to update the state-of-art knowledge on the field by conducting a systematic review and meta-analysis of OCD family and twin studies.

## Methods

### Search strategy and selection criteria

The present meta-analysis was conducted according to the Preferred Reporting Items for Systematic Review and Meta-Analysis (PRISMA) guidelines [57] and the study protocol was registered in PROSPERO (registration number: CRD42019118317).

The PECO strategy [58] was used to frame the systematic review procedure criteria, as follows. Participants: family members and/or twins of OCD probands; Exposure: family history of OCD; Controls: family members and/or twins of probands without OCD; and Outcome: OCD rates in relatives and/or twins of OCD probands.

For this systematic review and meta-analysis, we considered all studies that examined familial loading thorough family aggregation rates and/or twin resemblance up to September 30, 2021. The following databases were searched: CENTRAL (Cochrane Library); MEDLINE by PubMed (US National Library of Medicine); EMBASE; BVS (*Biblioteca Virtual em Saúde*); and OpenGrey (for gray literature). The search strategy was designed using DeCS headings and adapted to the terms for each database indexing vocabulary thesaurus (i.e., Medical Subject Headings [MeSH] for MEDLINE). No restrictions were placed on language or date of publication. Specific details of the search strategy for each database including the uniterms used are provided in Box S1.

A specific search strategy was used to locate previous reviews. The reference lists of all previously published reviews and meta-analyses were carefully screened, and included articles for full-text selection procedures, which were also scrutinized for additional relevant studies.

Studies were included if the following criteria were met: OCD diagnosis was assessed using standardized and validated instruments, or by a population database record (based on the *Diagnostic and Statistical Manual of Mental Disorders* [DSM-III or DSM-III-R, or DSM-IV, or DSM 5], or the *International Classification of Diseases, Eight, Ninth or 10th Revision* [ICD-8, ICD-9 or ICD-10] diagnostic criteria); the probands were diagnosed with OCD by direct interviews; relatives were directly interviewed; the study designs comprised cohort or case-control studies; and included a control group.

Review studies, case reports, expert consensus, letters to editor, opinion papers, segregation analysis studies, molecular genetic studies, studies reporting obsessive-compulsive personality disorder as the outcome, and animal model studies were excluded.

**Fig. 1:**
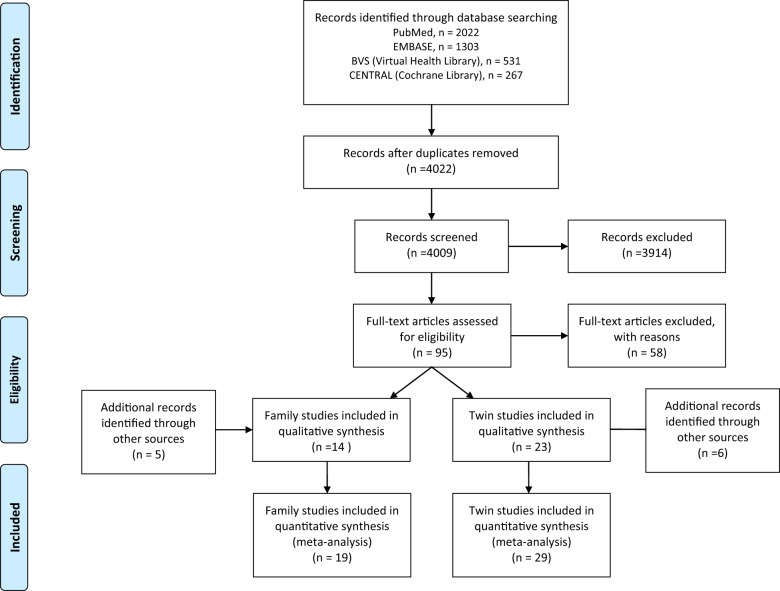
PRISMA flow diagram. From the 4022 studies screened, 19 family studies and 29 twin studies were selected for the systematic review and meta-analysis.

A flowchart illustrating the study search and selection process is presented in Fig. 1.

Two independent OCD experts (T.B.V. and L.M.) performed the screening procedure for the databases to determine which studies met the eligibility criteria. First, duplicate publications were screened and excluded (*n* = 101). Next, the screening process was applied to titles and abstracts (*n* = 4022), and potentially eligible full-text articles were selected (*n* = 95), followed by full-text review (T.B.V. and L.M.). Disagreements were discussed and resolved by consensus, or by consulting a third expert (M.C.R.). The Cohen’s kappa coefficient of agreement between the two reviewers was excellent (0.90). The screening process was performed using the Rayyan software [59].

During the study selection procedure, the reviewers concluded that all twin studies were based on community samples in which obsessive-compulsive symptoms (OCS) were not systematically assessed by standardized instruments and or direct interviews. This means that twin and population-based studies would have been excluded from the analysis, despite presenting extremely relevant information. Instead, we made the post hoc decision to wave this inclusion criterion for those studies (i.e., probands and FDRs were not directly interviewed).

From the 95 full-text records evaluated, 58 were excluded. A list of all the excluded studies after a full-text review and reasons for exclusion appears in the Supplement (Table S1). Of the remaining articles, 14 were family studies and 23 were twin studies. From the reference list search, five additional family and six additional twin studies were found and added to the meta-analysis. No studies were found in the gray literature.

The Newcastle-Ottawa Scale (NOS) [60] tool was used for the investigation of the risk of bias in case-control observational assessment. The NOS contains eight items, categorized into three domains: selection, comparability, and outcome/exposure. For each study type (i.e., family or twin), the items were adapted, and a series of response options were provided. A star system scoring was used, ranging from zero to nine stars. Details of the risk assessment of bias according to the NOS for each included study is provided in Table S2.

### Data analyses

For the coding process, a standardized data extraction form was used by the reviewers consisting of the following items: author(s) and year of publication; study location; sample recruitment procedure; inclusion and exclusion criteria applied for sample selection; OCD diagnostic criteria coding resource; and assessment tools used.

For family studies, it also included: case definition methods, including the adoption of the best estimate method (or not); matching procedures for proband comparison group selection; blindness of interviewer for proband status; number of probands, controls, and relatives with OCD (definite and subthreshold/probable); mean age of the proband and relative samples.

For family studies, the analysis unit was the FDR(s) of the OCD patients or controls, and the outcomes of interest were: (a) the familial recurrence rates of definite and probable/subthreshold OCD in FDRs of OCD compared with FDRs of control probands; and (b) the familial recurrence rates of definite and probable/subthreshold OCD in FDR of early-onset (before 18 years old) OCD probands compared with FDRs of late-onset (after 18 years old) OCD probands. We conducted these analyses three times: once for all studies, once for studies involving children/adolescents, and once for adults.

For each outcome of interest reported by ≥2 family studies, we performed a random-effects Mantel-Haenszel meta-analysis to derive pooled effect estimates. Because all data under analysis were dichotomous outcome variables, we summarized the effects using odds ratio (OR) and their 95% confidence intervals (CIs).

For twin studies, the number of MZ and DZ pairs and the twin resemblance correlations according to zigozity were systematically extracted. When the studies only reported separate correlations for males and females, we transformed the correlation coefficients to Fisher z-values (see below), averaged them, and back-transformed the resulting z-value to a correlation coefficient.

For the twin studies, the analysis unit was the twin pairs. The outcome of interest was the correlation of OCS in monozygotic compared with dizygotic twins. We tested two hypotheses: (a) that the OCS correlation in monozygotic twins is equal to the OCS correlation in dizygotic twins; and (b) that the OCS correlation in monozygotic twins is the double of the OCS correlation in dizygotic twins. We conducted these analyses considering all studies, including only the children/adolescents and only adults.

For each outcome of interest reported by ≥2 twin studies, we transformed the correlation coefficients to Fisher Z values and performed a random-effects meta-analysis to derive pooled effect estimates. This transformation was beneficial because the standard error of a correlation depends on the correlation itself, making larger correlations appear more precise and thus receiving more weight. In contrast, the Fisher transformation only depends on the sample size. We conducted these analyses twice: once assuming that the standard error was that of the Pearson correlation and once assuming that it was that of the tetrachoric correlation. The latter assumes that the presence of OCS represents latent variables that follow a bivariate normal distribution. To use the standard error of the tetrachoric correlation, we first estimated the number of concordant and discordant twin pairs for the presence of OCS according to a 2.3% lifetime prevalence of OC [61] then using the “polychor” function to derive the standard error [62] and finally calculating the “effective” sample size to perform the meta-analysis, as described in Polderman et al. [63].

Finally, we used tetrachoric correlations to estimate the A (additive genetics), the C (shared environmental) and the E (non-shared environmental) components based on the following definitions:

$$r_{MZ} = {{{\mathrm{A + C}}}}$$

$$r_{DZ} = 0.5{{{\mathrm{A}}}} + {{{\mathrm{C}}}}$$

$$var = {{{\mathrm{A}}}} + {{{\mathrm{C}}}} + {{{\mathrm{E}}}}$$

We measured the heterogeneity between studies with the *I*^2^ statistic, which describes the percentage of the variability in effect estimates attributable to heterogeneity. We accepted *I*^2^ values <50%. When the *I*^2^ value exceeded this value, the studies were excluded one-by-one from the analyses to identify and analyze the outlier.

All statistical analyses were conducted using R with the packages “meta” (for meta-analysis) [64] and “polycor” (for tetrachoric correlations) [62].

## Results

### Family studies

Nineteen eligible family studies were included in the meta-analysis (Table 1). Almost all exclusions of family studies were due to the absence of a control group or the lack of an OCD diagnosis confirmation using standardized instruments and/or direct interviews of probands or relatives (see Table S1). Four studies (comprising three samples) assessed families of child/adolescent probands [17, 20–22]. Another 14 studies, comprising nine samples, reported data on family risk for adult OCD probands [4, 5, 16, 18, 23–29, 65–67]. One study did not compare OCD familial risk between case and control probands (probands without OCD) but were included because they compared OCD familial risk between early and late-onset OCD probands [68]. Finally, from the nineteen included publications, 18 studies [4, 5, 16–18, 20–29, 65–67] (comprising twelve different samples) were considered for the main analyses.

**Table 1:** OCD family studies.

			OCD and comorbid disorders assessment tools		Diagnosis criteria and control				Sample size						Diagnosis in first-degree relatives					
Author/year	Country	Proband age inclusion criteria	Proband	Relatives	Blind to proband status (y/n)	Best estimate diagnosis procedure (y/n)	Diagnostic criteria	Controls matched for gender and age (y/n)	Proband				First-degree Relatives		Definite OCD		Probable OCD		Definite/Probable OCD	
									Case	Control	Case	Control	Case	Control	Case	Control	Case	Control		
									*n*	Mean age (SD)	*n*	Mean age (SD)	*n*	*n*	*n*	*n*	*n*	*n*	*n*	*n*
*Child proband*																				
Reddy et al. [20]	India	Juvenile proband (<=16 y)	DICA-R; CV-LOI; CY-BOCS. questionnaire for tic	Same if child; SCAN, LOI, Y-BOCS, questionnaire for tic disorder, if adult.	n	y	DSM-III-R	y	35	13.6 (2.4)	34		106	94	4	0				
Hanna et al. [21] Hanna et al. [22]	USA	Adolescent proband (10–17 y)	K-SADS-E; STOBS	SCID-III-R, Y-BOCS, FISC; STOBS	y	y	DSM-III-R	n	35	13.7 (2.4)	17	12.4 (1.8)	102	39	23 (22.5)	1 (2.6)			28 (27.4)	1 (2.6)
Rosario Campos et al. [17]	USA	Children and adolescent proband (<=18 y)	STOBS; Y-BOCS; YGTSS; KSADS; SCID	Same	y	y	DSM-IV	y	106	11.9 (3.03)	44	11.2 (2.06)	325	140	62 (22.7)	1 (0.9)			79 (29.2)	3 (2.4)
*Adult proband*																				
McKeon and Murray [65]	USA	Adult proband	SADS-L	SADS-L; SAP	n	–	Research Diagnostic Criteria (RDC)	y	50	Not reported	50	Not reported	149	151	1	1				
Black et al. [66]; Black et al. [4] *Iowa family study of OCD	USA	Adult proband	SADS; Y-BOCS; NIMHOCS; Hamilton Depression Rating Scale	DIS; SIDP; FHRDC	y	–	DSM-III	y	32	37.9 (10.9)	33	38.4 (10.2)	168	184	1 (0.6)	1 (0.5)				
Pauls et al. [16]; Carter et al. [29]	USA	Adult proband	STOBS; DIS; K- SADS-E; Y-BOCS	Same	y	y	DSM-III-R	n	100	33.6 (12.1)	33		466	113	47	2	35	2	82	4
Bienvenu et al. [67]; Nestadt et al. [18]; Grados et al. [23]; Nestadt et al. [24] *Johns Hopkins OCD Family Study	USA	Adult proband (>=18 y)	SADS-LA; Y-BOCS; LOI; FICS; YGTSS	All+ K-SADS, CY-BOCS, if child	y	y	DSM-IV	y	80	37 (18–88)	73	39 (18–79)	326	297	38 (11.7)	8 (2.7)			53 (16.3)	17 (5.7)
Black et al. [25]	USA	Adult proband	SCID- IV; Y-BOCS; SIDP-IV; FAD	*Offsprings (child between ages 7 and 18 years) DICA, CBCL for children	y	y	DSM-IV	y	21	39.5 (6.4)	22	38.2 (5.6)	43	35	10 (23)	1 (3)	13 (30)	8 (23)		
Fyer et al. [26]	USA	Adult proband	SADS-LA, FISC	Same	y	y	DSM-III-R	y	72	34.8 (11)	32	43^.^6 (15)	179	112	11 (6.2)	0 (0)			15 (8.4)	0
Lipsitz et al. [27]	USA	Adult proband	SADS-LA, FISC	Same	y	y	DSM-IV	y	57	37.2 (13)	41	42.6 (13)	112	115	4 (3.6)	2 (1.7)			6 (5.4)	2 (1.7)
Grabe et al. [28]	Germany	Adult proband (>=18)	SADS-LA	Same	y	y	DSM-IV	n	105	38.3/44.8	70	42.1 (16.4)	343	247	22 (6.4)	3 (1.2)	19	7		
Bienvenu et al. [5] *Johns Hopkins OCD Family Study *OCD Collaborative Genetics Study	USA	Adult proband	SADS-LA; SCID-IV	All those + K-SADS	y	y	DSM-IV	y	382	Not reported	73	Not reported	974	233	524 (55)	16 (7)				
*Nationalwide register-based studies*																				
													Full sibling (OR)		Parent (OR)		Offspring (OR)			
Mataix-Cols et al. [7]	Sweden	all psychiatric patients treated in Sweden (1969–2009)	Swedish National Patient Register	Same	n	n	ICD-10	n	24768		13589819		5.03 (4.49–5.64)		4.70 (4.09–5.40)		4.56 (3.97–5.24)			
													Full sibling (OR)		Maternal (OR)		Paternal (OR)		Offspring (OR)	
Steinhausen et al. [8]	Denmark	All individuals born between 1952 and 2000	Danish Psychiatric Central Research Register	same	n	n	ICD-8 ICD-10	y	2057		6055		6.19 (6.65–10.49)		11.21 (3.14040.04)		34.04 (4.42–262-39)		4.54 (1.28–16.13)	
													Full sibling (RRR)							
Browne et al. [9]	Denmark	Any individuals who had contact with the national health care system (1969–1995)	Danish Psychiatric Central Register; Danish National Hospital Register	same	n	n	ICD-8 ICD-10	n	6191		1734668		4.89 (3.45–6.93)							
													Full sibling (OR)		Maternal half siblings (OR)		Paternal half siblings (OR)		Twins (OR)	
Brander et al. [10]	Sweden	Any individuals born between 1967 and 2007	Swedish national registers	same	n	n	ICD-8 ICD-9 ICD-10	n	22232		4063135		10.63 (7.92–14.27)		3.72 (1.92–7.20)		0.30 (0.04–2.12)		25.19 (9.20–68.98)	
													Full sibling (RRR)		Maternal half siblings (RRR)		Paternal half siblings (RRR)			
Mahjani et al. [11]	Sweden	Any individuals born between 1982 and 1990	Swedish Medical Birth Register	same	n	n	ICD-10	n	7184		815659		4.82 (4.03–5.62)		1.85 (0–2.37)		1.09 (0–1.96)			
													Full sibling (RR)		Parents (RR)		Offspring (RR)		Twins (RR)	
Huang et al. [12]	Taiwan	All individuals diagnosed with OCD between 1 January 2001 and 31 December 2010	Taiwan National Health Insurance Database	same	n	n	ICD-9	n			23258175		8.95 (8.44–9.49)		7.64 (7.10–8.23)		7.18 (6.65–7.75)		60.76 (49.12–75.16)	

*DICA-R* Diagnostic Interview for Children and Adolescents–Revised, *CV-LOI* Children’s Version of Leyton’s Obsessional Inventory, *CY-BOCS* Children’s Version of the Yale-Brown Obsessive Compulsive Scale, *SCAN* Schedule for Clinical Assessment in Neuropsychiatry, *LOI* Leyton’s Obsessional Inventory, *Y-BOCS* Yale-Brown Obsessive Compulsive Scale, *FISC* Family Informant Schedule and Criteria, *K-SADS-E* Schedule for Affective Disorders and Schizophrenia for School Age Children-Epidemiologic Version, *SCID-III-R* Structured Clinical Interview for DSM-III-R, *STOBS* Schedule for Tourette and Other Behavioral Syndromes, *YGTSS* Yale Global Tic Severity Scale, *SCID* Structured Clinical Interview for DSM.

The * shows the study group which the study was related to.

**Fig. 2:**
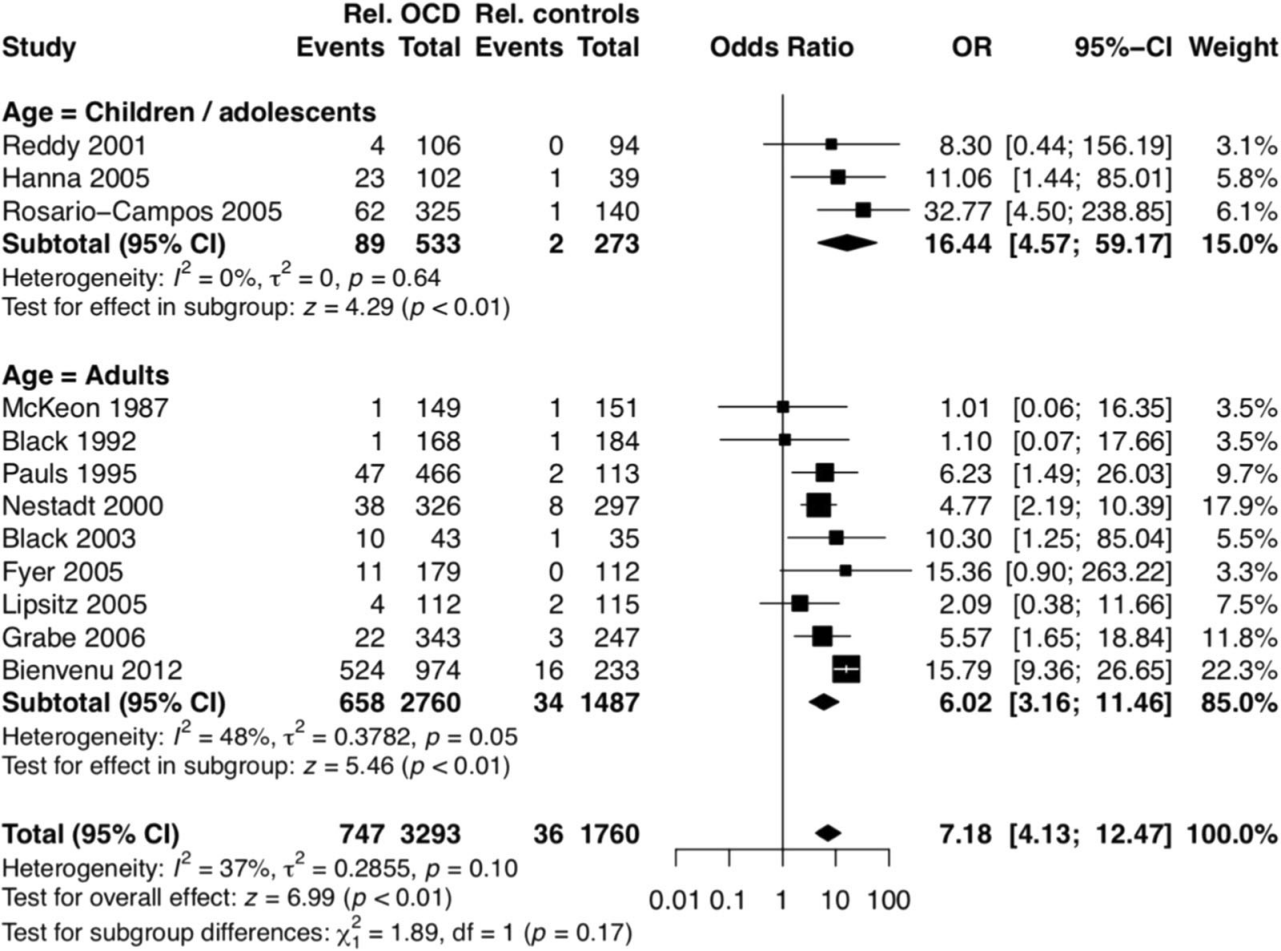
Definite OCD prevalence. First-degree relatives of OCD probands had odds ratio of 7.18 (95% CI 4.13–12.47, *p* < 0.00001) for definite OCD in comparison to controls.

Data from 5053 directly interviewed FDRs of 176 child and adolescent and 899 adult OCD probands and 522 control probands (95 from children/adolescents and 427 from adult samples) were pooled for meta-analysis. The analyses combining pediatric and adult probands showed that FDRs of OCD probands had higher risks for definite OCD (OR = 7.18, 95% CI 4.13–12.47, *p* < 0.00001) than the FDRs of controls (Fig. 2).

**Fig. 3:**
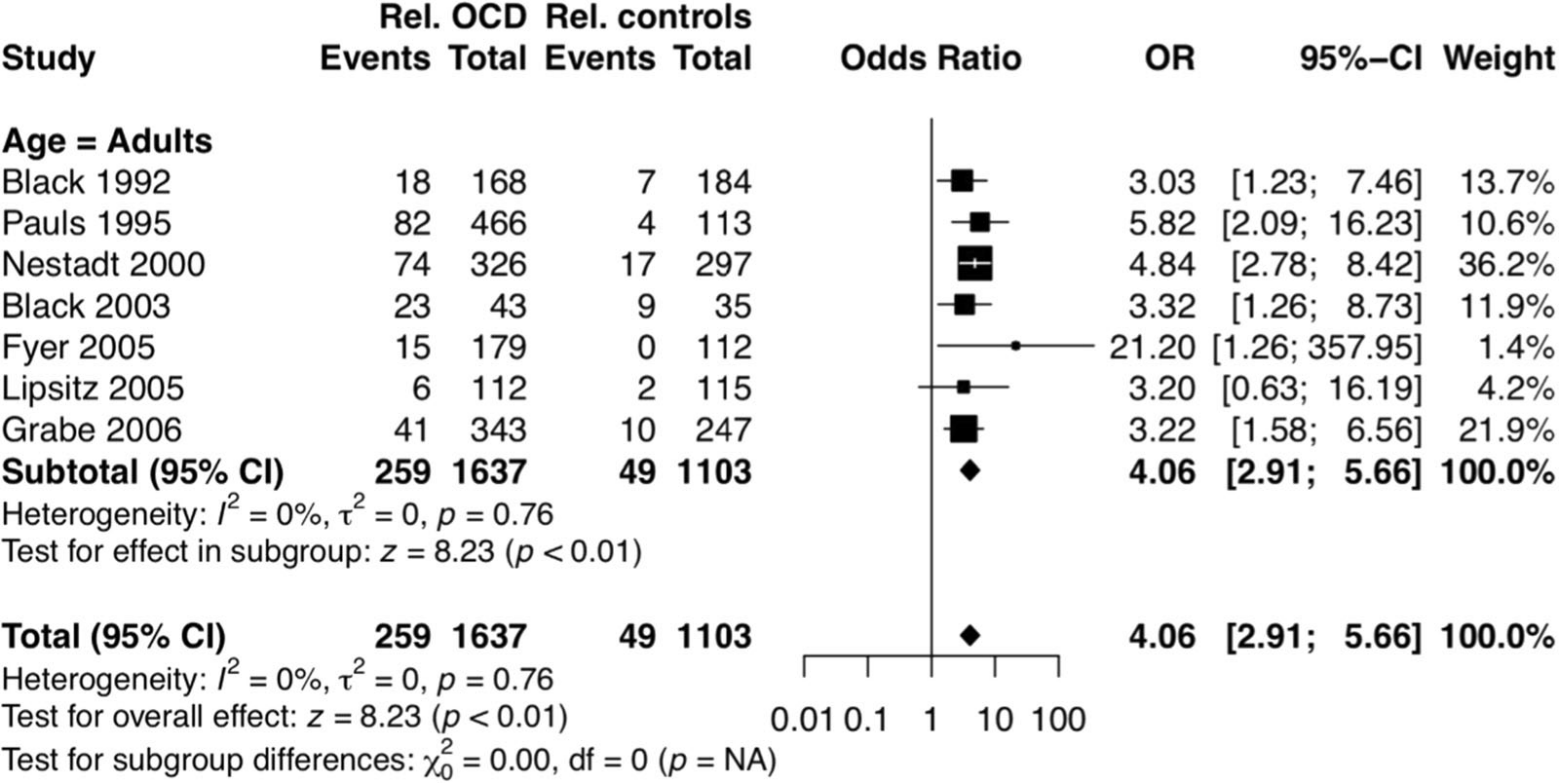
Definite/subthreshold OCD prevalence. First-degree relatives of definite and subthreshold OCD probands had 4.6-times higher risk of OC symptoms compared to controls.

The comparison between pediatric and adult studies indicated that OCD was significantly more familial in children/adolescents than in adults. First-degree relatives of the child and adolescent OCD probands had a 16 times higher risk of definite OCD compared to the control FDRs (OR = 16.44, 95% CI 4.57–59.17, *p* < 0.00001). The FDRs of adult OCD probands had an approximately 6 times higher risk of definite OCD compared to the control FDRs (OR = 6.02, 95% CI 3.16–11.46, *p* < 0.00001) (Fig. 3).

For the above analyses, the degree of heterogeneity among the studies was acceptable (overall *I*^2^ = 37%; adult probands, *I*^2^ = 48%; child and adolescent probands, *I*^2^ = 0).

Regarding definite and subthreshold OCD, the pooled data among adult samples revealed slightly lower familial loading (OR = 4.06, 95% CI 2.91–5.66, *p* < 0.00001) than the analyses including only definite OCD. There was no heterogeneity between the studies pooled for this analysis (*I*^2^ = 0%).

**Fig. 4:**
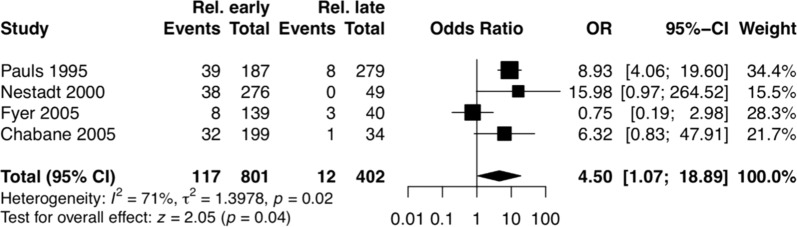
Age of onset. Only four samples remained for OCD family recurrence rate statistical analysis. The studies showed a high heterogeneity for the analyzed outcome.

Data on the occurrence of definite or definite/subthreshold OCD in FDRs of probands considering the age of symptom onset were reported for eight different samples in 12 publications [5, 16, 18, 23, 25–29, 66, 68]. However, eight studies did not present the number of relatives included in their studies, remaining only four samples for the statistical analyses [16, 18, 26, 68]. Together, the studies showed a high heterogeneity for the analyzed outcomes (definite OCD, *I*^2^ = 71%; definite/subthreshold OCD, *I*^2^ = 95%) (Fig. 4).

The tic-related OCD family aggregation analyses were not reported because of the insufficient number of studies included or the high heterogeneity of the extracted data.

### Risk of bias

The Newcastle-Ottawa Scale (NOS) scores of the included family studies were generally high, indicating low risk of bias (Tables S2). The only exceptions were the studies by [65] and [20], which lacked an interviewer blinding procedure regarding proband/relative status, and [13, 69], and [68], which did not use the best estimate diagnosis method and lacked control samples.

### Twin studies

**Fig. 5:**
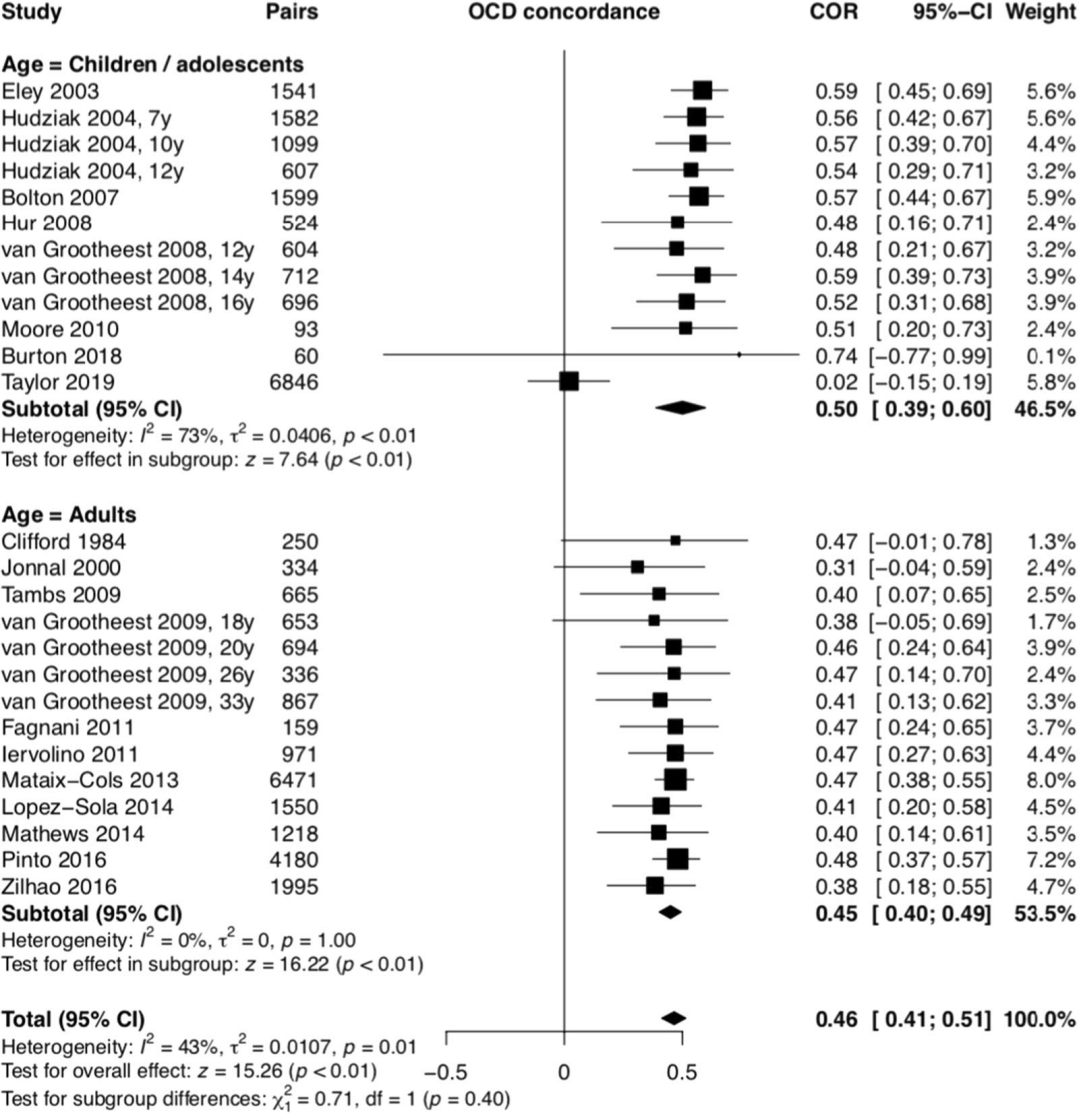
Monozigotic twin OCS resemblance. OCS coccurrence rate in monozigotic twin pairs was about 47%.

**Fig. 6:**
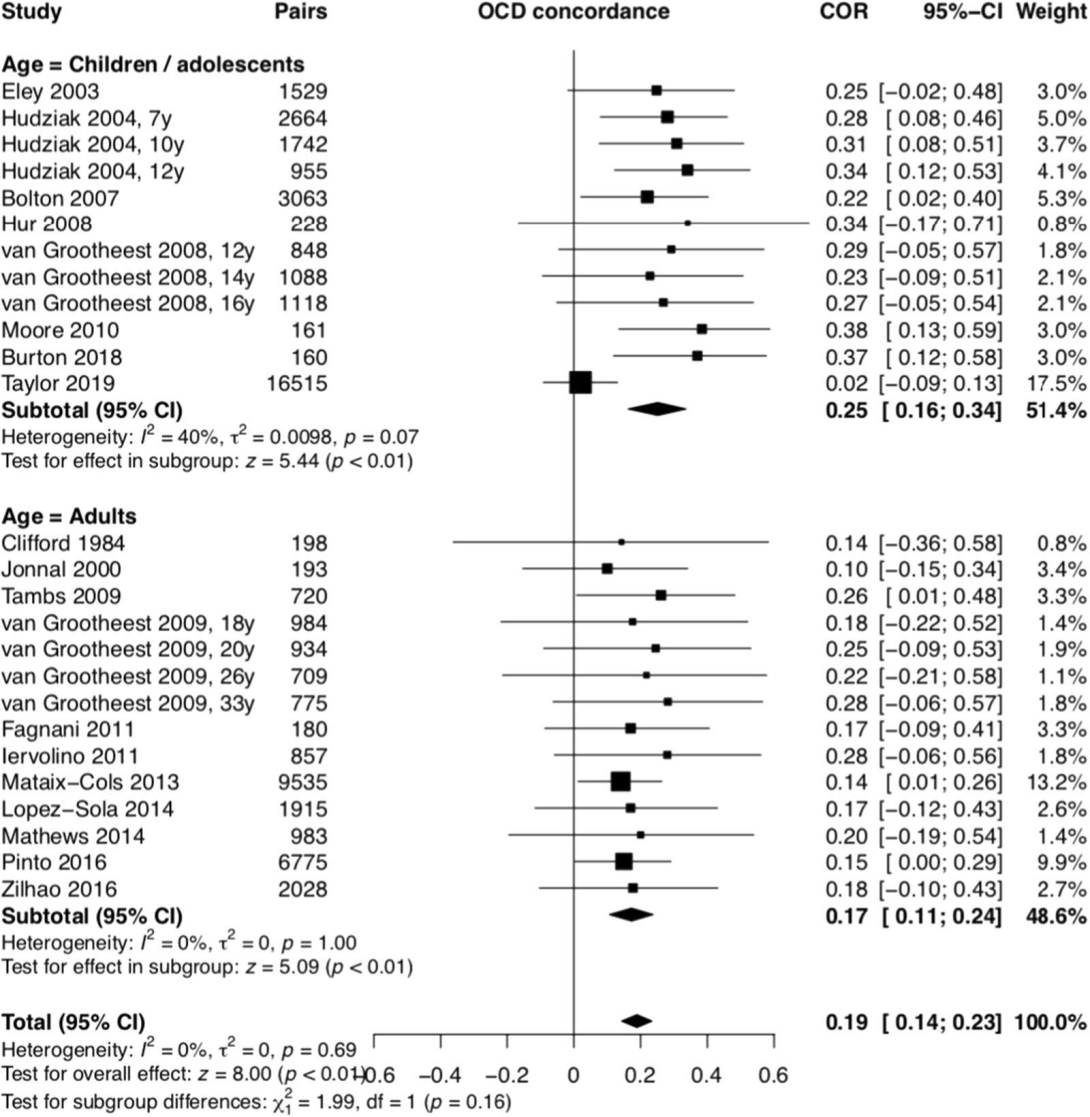
Dizogotic twin OCS resemblance. OCS cooccurence rate between dizigotic twin pairs was about 23%.

Twenty-nine twin papers were included in the metanalysis [7, 30–56, 70] (Table 2), comprising 26 different samples. These studies involved self-report of symptoms using a range of validated questionnaires. Table 2 and Figs. 5 and 6 depict the MZ and DZ twin correlations from 29 twin studies (eight with child/adolescent samples) and the metanalytic summary estimates. The meta-analysis revealed similar correlations across the age subgroups and the total sample and that MZ twins had significantly higher correlations for OCD when compared to DZ twins (children/adolescents: *r*_MZ_ = 0.52, 95% CI 0.37–0.65, *p* < 0.01; *r*_DZ_ = 0.27; 95% CI, 0.18–0.36, *p* < 0.01; adults: *r*_MZ_ = 0.43, 95% CI 0.40–0.45, *p* < 0.01; *r*_DZ_ = 0.20; 95% CI, 0.17–0.22, *p* < 0.01; total: *r*_MZ_ = 0.47, 95% CI 0.40–0.54, *p* < 0.01; *r*_DZ_ = 0.23; 95% CI, 0.19–0.27, *p* < 0.01). The use of the standard error of the tetrachoric correlation did not generate significant changes in the above results.

**Table 2:** OCD twin studies.

Author/year	Country	Inclusion age criteria	Twin pairs		Assessment tools	Twin correlations by zigozity							Variance component estimates based on ACE model coefficient, 95% CI		
			MZ *n*	DZ *n*		MZ	MZM	MZF	DZ	DZM	DZF	DOS	Additive genetic (A^2^)	Shared environment (C^2^)	Non-shared environment (E^2^)
Clifford et al. [30]	*UK*	Adult	250p	198p	LOI	–	0.50	0.44	–	0.22	0.13	0.11	–	–	–
Jonnal et al. [31] van Grootheest et al. [70]	*USA* *Virginia Twin Registry	Adult	331p	193p	PADUA	0.34 (C) 0.28 (O)	–	–	0.14 (C) 0.06 (O)	–	–	–	0.36	0.0	0.64
Eley et al. [33]	*UK* *Twins Early Development Study (TEDS)	Children (4 y)	2359	6046	OCB (not standardized instrument)		0.59	0.58		0.19	0.28	0.26	0.65 (0.61–0.69)		0.37 (0.31–0.39)
Hudziak et al. [34]	Netherlands * Netherlands Twin Registry (NTR)	Children	*7* *y* 1582p *10* *y* 1099p *12* *y* 607p	2664 1742 955	CBCL-OCS	– – –	0.55 0.59 0.57	0.57 0.54 0.50	– – –	0.31 0.35 0.30	0.21 0.22 0.40	0.30 0.33 0.33	0.56 0.55 0.36	0.0 0.0 0.16	0.56 0.55 0.36
Bolton et al. [35] Bolton et al. [55]	UK *TEDS	Children (6 y)	1599p	3063p	ADIS-C/P	0.57 (0.24–0.80) 0.67 (0.22–0.92)	–	–	0.22 (−0.22–0.43) 0.38 (0.07–0.64)	–	–	–	0^.^29 (0^.^00–0^.^68) 0.55 (0.08–0.89)	0^.^18 (0^.^00–0^.^48) 0.11 (0.00–0.38)	0^.^53 (0^.^28–0^.^80) 0.34 (0.09–0.65)
van Grootheest et al. [56]	Netherlands *NTR	Adolescents and adult	1584	7662	YARS-OCS	–	0.44	0.50	–	0.13	0.26	0.21	–	–	–
van Grootheest et al. [32]	Netherlands *NTR	Adolescents	*12* *y* 604 *14* *y* 712 *16* *y* 696	848 1088 1118	YARS-OCS, PADUA ABBR	– – –	0.50 0.57 0.45	0.45 0.60 0.58	– – –	0.38 0.17 0.30	0.36 0.30 0.33	0.21 0.22 0.22	0.27 0.57 0.54	0.18 0.00 0.00	0.54 0.43 0.46
Hur and Jeong [37]; Hur [36]	Korea *South Korean Twin Registry (SKTR)	Juvenile (13–24 y)	524p	228p	MOCI		0.56 (0.45–0.65)	0.39 (0.30–0.48)		0.24 (−0.1–0.46)	0.36 (0.14–0.56)	0.38 (0.20–0.53)	0.69 (0.64–0.73)	–	0.73 (0.69–0.77)
Tambs et al. [38]	Norway *Norwegian Institute of Public Health Twin Panel (NIPHTP)	Adults (28 y)	*665p*	720p	CIDI	–	0.45	0.35	–	0.20	0.32	–	0.36 (0–0.65)	0.31 (0–0.49)	0.70 (0.59–0.80)
van Grootheest et al. [39]	Netherlands *NTR	Adults	*18* *y* 653p *20* *y* 694p *26* *y* 336p 33 y 867p	984 934 709 775	YARS-OCS, PADUA ABBR	– – – –	0.41 0.39 0.46 0.37	0.35 0.53 0.47 0.44	– – – –	0.10 0.24 0.11 0.35	0.25 0.25 0.32 0.21	– – – –	– – – –	– – – –	– – – –
Moore et al. [40]	Ireland	Children (12 y)	93p	161p	LOCI-CV		0.678	0.300		0.361	0.406				
Fagnani et al. [41]	Italy	Adult (23–24 y)	159p	180p	SCL-90	0.47			0.17				0.451 (0.099–0.586)	0.000 (0.000–0.267)	0.549 (0.415–0.711)
Iervolino et al. [42]	UK *TwinsUK adult twin register	Adults women (17–86 y)	2720	1635	OCI-R	0.47 (0.40–0.53)	–	–	0.28 (0.19–0.36)	–	–	–	0.380 (0.187–0.526)	0.090 (0.000–0.252)	0.530 (0.472–0.595)
Lahey et al. [43]	USA *Tennesse Twin Study	Adolescents (6–17 y)	Not described		CAPS	0.64			0.32				0.640 (0.457–0.684)	0.000 (0.000–0.166)	0.360 (0.316–0.411)
Mataix-Cols et al. [7]	Sweden *STAGE study	Adults (20–47 y)	6471	9535	Clinical interview based on ICD-8,9,10	–	0.45	0.49	–	0.20	0.12	0.12	0.47 (0.40–0.52)	0.00 (0.00–0.05)	0.53 (0.48–0.58)
López-Solà et al. [44] López-Solà et al. [45]	Australia *Australian Twin Registry (ATR)	Adults (18–45 y)	1281	1214	OCI-R	0.41 (0.34, 0.48)	0.40 (0.27, 0.51)	0.42 (0.32, 0.51)	0.17 (0.06, 0.26)	0.16 (0.03, 0.33)	0.23 (0.09, 0.36)	0.06 (0.12, 0.23)	0.39 (0.18–0.45)	0.00 (0.00–0.18)	0.61 (0.55–0.68)
Mathews et al. [46]	Netherlands *NTR	Adult	1218	983	PI-ABBR	0.40	0.40	0.40	0.20	0.20	0.20	0.20	0.376	0.022	0.601
Monzani et al. [47]	UK *TwinsUK adult twin register	Adult women (16–90 y)	3042	2367	OCI-R	–	–	0.52	–	–	0.21	–	0.48 (0.42–0.53)	–	0.52 (0.47–0.58)
Zilhão et al. [48]	Netherlands *NTR	Adult	4566	4714	PI-ABBR	0.367 (0.327–0.406)	0.400 (0.324–0.468)	0.359 (0.308–0.400)	0.119 (0.867–0.151)	0.130 (0.090–0.169)	0.084 (0.036–0.131)	0.157 (0.083–0.228)	–	–	–
Krebs et al. [49]	UK *TEDS	Children and adolescent (cohort)	*4* *y* 5072 *7* *y* 5202 *9* *y* 2421 *16* *y* 3497	9671 9216 4103 9647	ARBQ	– – – –	– – – –	– – – –	– – – –	– – – –	– – – –	– – – –	0.59 (0.57–0.61) 0.61 (0.57–0.63) 0.54 (0.46–0.61 0.33 (0.26–0.41)	0.00 (0.00–0.01) 0.02 (0.01–0.04) 0.14 (0.09–0.21) 0.25 (0.19–0.31)	0.41 (0.38–0.43) 0.38 (0.36–0.40) 0.32 (0.29–0.35) 0.42 (0.39–0.45)
Pinto et al. [50]	Sweden *STAGE study	Adults (19–47 y)	8360	13551	Clinical interview based on ICD-8,9,10	0.48 (0.48–0.52)	–	–	0.15 (0.10–0.18)	–	–	–	0.55	–	0.63
Taylor et al. [51]	Canada	Adult (17–81 y)	167	140	OCI-R	–	–	–	–	–	–	–	0.53	–	0.47
Zilhão et al. [52]	Netherlands *NTR	Adult	3990	4057	PI-ABBR	0.384	0.379	0.386	0.177	0.197	0.139	0.214	0.373	–	0.627
Taylor et al. [53]	Sweden *Child and Adolescent Twin Study (CATS)	Child and adolescent	*Not described*		Parent-rated A-TAC compulsion module (9 and 12 y); self-rated BOCS (18 y)	0.02			0.02					–	0.627
Burton et al. [54]	Canada	Adolescent (6–18 y)	*60p*	160p	TOCS CBCL-OCS	0.74 (0.60–0.83) 0.62 (0.44–0.75)	–	–	0.37 (0.23–0.50) 0.34 (0.20–0.47)	–	–	–	0.74 (0.63–0.82) 0.56 (0.40–0.68)		0.26 (0.18–0.37) 0.44 (0.32–0.60)

*MZ* monozigotic, *DZ* dizogotic, *MZM* monoigotic male, *MZF* monozygotic female, *DZM* dizygotic male, *DZF* dizygotic female, *DOS* dizygotic opposite sex, *HRS-SR* Hoarding Rating Scale-Self-Report, *PI-ABBR* Padua Inventory Abbreviated Revised, *OCI-R* Obsessive Compulsive Inventory-Revised, *ARBQ* Anxiety-Related Behavior Questionnaire, *CAPS* Child and Adolescent Psychopathology Scale.

The * shows the study group which the study was related to.

**Fig. 7:**
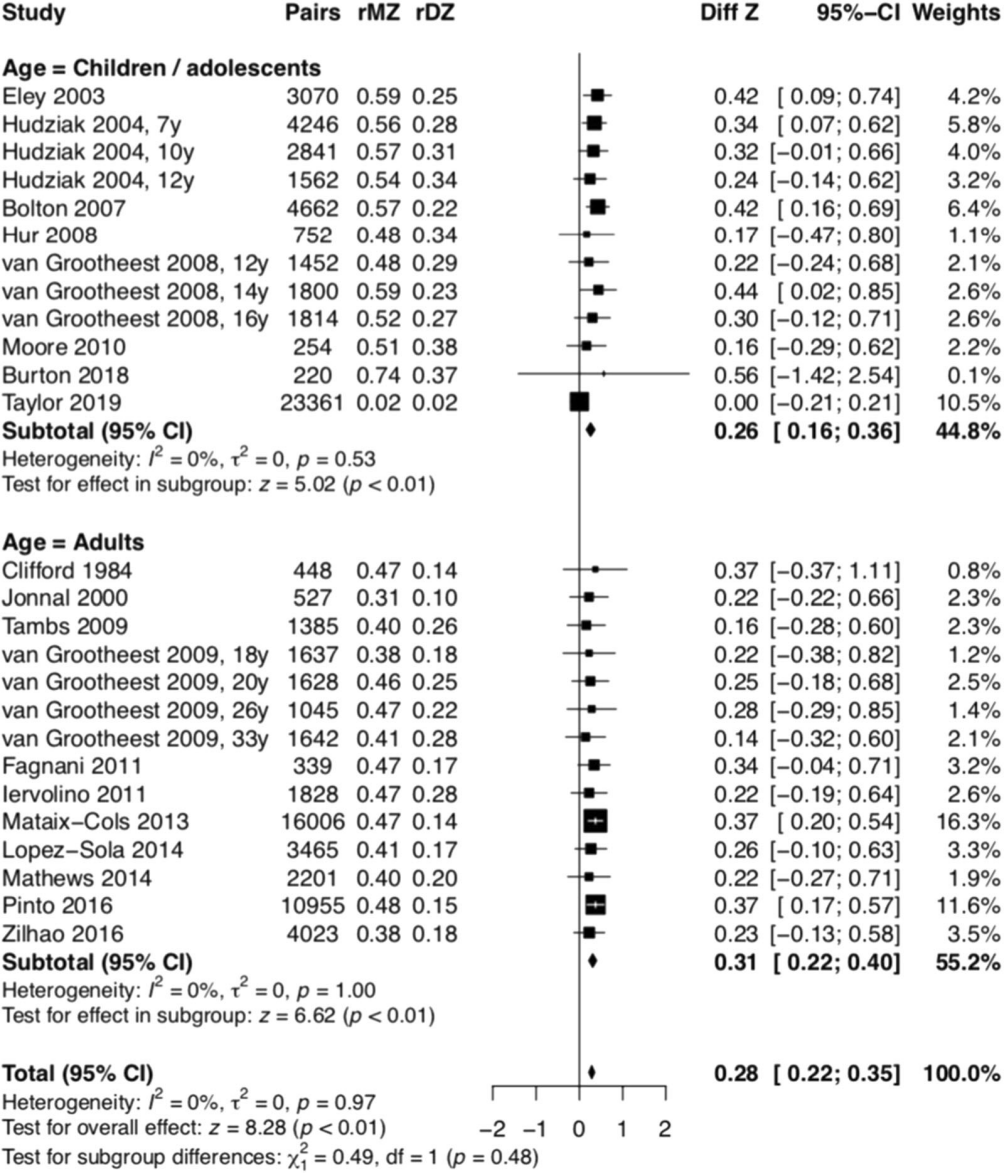
Fisher *Z*-score between monozigotic and dizogotic twins. MZ twins has considerably higher correlations for OCS than DZ twins.

The Fisher z-score analysis confirmed that MZ twins showed considerably higher correlations for OCS than DZ twins (children/adolescents: Diff Z = 0.30, 95% CI 0.17–0.42, *p* < 0.01; adults: *Diff Z* = 0.26, 95% CI 0.21–0.31, *p* < 0.01; total: *Diff Z* = 0.28, 95% CI 0.21–0.34, *p* < 0.01) (Fig. 7). Based on the tetrachoric correlations, we estimated that A = 0.46, C = 0, and E = 0.54 for the total sample; A = 0.50, C = 0, E = 0.50 for children/adolescents; and A = 0.45, C = 0, and E = 0.55 for adults.

Of note, there was a high heterogeneity index across the twin analyses. However, it is important to mention that despite the high heterogeneity, almost all results were statistically significant in the same direction.

### Population-based cohorts (post hoc)

Six large nationwide register-based cohorts [7–12] did not meet our initial inclusion criteria but, given their superior statistical power and relevance, they were included in the current paper and their results are narratively described below.

From a cohort of more than 13.5 million people who were born or lived in Sweden between 1969 and 2009, Mataix-Cols et al. [7] found that the FDRs of the 24,768 individuals with OCD were more likely to also have OCD (OR = 5.03, 95% CI = 4.49–5.64 for siblings; OR = 4.70, 95% CI = 4.09–5.40 for parents; and OR = 4.56, 95% CI = 3.97–5.24 for the offspring); that this risk decreased proportionally to the degree of genetic relatedness; and that the risk tended to be higher amongst FDR of early-onset OCD individuals.

Brander et al. [10] studied the same population between 1967 and 2007. They reported that full siblings of individuals with tic-related OCD (HD = 10.63, 95% CI = 7.92–14.27) had a higher risk for OCD than the relatives of non-tic-related OCD (HD = 4.52, 95% CI 4.06–5.02).

Mahjani et al. [11] also analyzed the OCD familial risk in the Swedish population. The cohort enrolled 822,843 people born between 1982 and 1990, of which 7184 (0.87%) were diagnosed with OCD. The relative recurrence risks (RRRs) confirmed the OCD familial pattern (RRR = 4.82, 95% CI = 4.03–5.62 for full siblings; RRR = 1.85, 95% CI = 0–3.27 for maternal half-sibling; RRR = 1.09, 95% CI = 0–1.96 for paternal half-siblings; RRR = 1.85, 95% CI = 1.29–2.41 for maternal cousins; and RRR = 1.59, 95% CI = 1.13–2.08 for other cousins).

Steinhausen et al. [8] investigated a population sample from 1969 until 2009 in Denmark. The data indicated that an early-onset of OCS (*N* = 2057) significantly increased the risk of having OCD in the FDRs compared with controls (*N* = 6055) (OR = 34.4, 95% CI = 4.42–262.39 for paternal OCD; OR = 11.21, 95% CI = 3.14–0.04 for maternal OCD; OR = 6.19, 95% CI = 3.65–10.49 when a sibling had OCD; and an OR = 4.54, 95% CI = 1.28–16.13 if one of the children had OCD).

Browne et al. [9] analyzed data from the same Danish population (born from 1980 to 2007). The individuals with an older sibling (RRR = 4.89, 95% CI = 3.45–6.93) or a parent (RRR = 6.25, 95% CI = 4.81–8.11) diagnosed with OCD exhibited higher risk of having OCD.

Finally, Huang et al. [12] investigated the risk of OCD among 89,500 FDRs of OCD patients in Taiwan from 2001 until 2010. Compared to the general population, the FDRs of OCD patients had a higher risk of OCD (RR = 8.11, 95% CI = 7.68–8.57). More specifically, the relative risks were: RR = 7.64, 95% CI = 7.10–8.23 for parents; RR = 7.18, 95% CI = 6.65–7.75 for the offspring; RR = 8.95, 95% CI = 8.44–9.49 for siblings; and RR = 60.76, 95% CI = 49.12–75.16 for twins.

## Discussion

The current meta-analysis included all OCD family and twin studies published until September 2021. The results update and extend the findings of the previous meta-analysis published more than 20 years ago [19]. The main findings were that OCD is highly familial, particularly in children and adolescents; that the heritability of OCS in twin samples is approximately 0.5; and that the higher OCS correlations between MZ twins were mainly due to additive genetic or to non-shared environmental components. These results are relevant for future genetic and clinical studies and reinforce the need for the development of specific guidelines for the screening of OCS in the FDRs of OCD subjects and the early referral for treatment when needed.

According to the 18 OCD family studies included in the analyses, OCD was 7.2 times more frequent in OCD families, when compared to control families. These estimates are almost twice higher than those from the last meta-analysis published in 2001 [19]. One conceivable explanation for these different ratings could be the fact that the current study included studies with larger samples, interviewed with validated assessment tools and based on reliable diagnostic criteria. Furthermore, considering the secrecy characteristic of OCD, the higher rates may be due to the fact that the current analyses included subjects that were directly interviewed.

Of note, the OCD rates among control relatives (2.3%) were very similar to the lifetime prevalence rates in the general population, ranging from 0.7% to 3% [61]. These findings suggest that the current results may be generalized to other samples and reinforce the robustness of the current estimates.

Additional analyses of very large population-based studies, primarily conducted in Scandinavian countries and Taiwan, support the estimates from the family studies [7–12]. In line with the studies that did meet our inclusion criteria, the risk for OCD in these population-based studies varied from 4.7 [7] to 7.64 [12] for parents, 4.82 [11] to 8.95 [12] for full siblings, and 4.54 [8] to 8.95 [12] for the offspring. Because these population studies had superior statistical power and less risk of selection bias, we conclude that the familial risk estimates are generalizable to the general population.

The twin studies demonstrated that OCD, or at least its dimensional representation, is not only familial but also heritable, with twin correlations ranging from 0.52 and 0.43 in MZ twins compared to 0.27 and 0.20 in DZ twins (in children and adult samples, respectively). These findings are in line with previous reports [1, 71, 72], and indicate that both genetic and environmental characteristics are important in the etiology of OCS. The analyses of the specific roles of additive genetic effects (A) and non-shared environment (E) components of the ACE model in the etiology od OCD revealed that our findings are in line with previous results [1] with each accounting for 46% and 54% of the variance, respectively. Interestingly, single-nucleotide polymorphisms -based heritability of OCD is still considerably lower, in the region of 30% [73], which indicates that further research is needed to understand the “missing heritability”. It is plausible to assume that while the majority of inherited liability for OCD is due to common genetic variation, rare variation may also contributes to some extent. Thus, future genetic studies should focus on common as well as rare genetic variants as a way to capture more of the unexplained phenotypic heritability.

Notably, the shared environment component (C) did not have any contribution to the etiology of OCS in this study. Taylor, 2011 [1] have also previously reported that the shared environment has a weak contribution to the OCS phenotypic variance. This finding is particularly relevant to the clinical field because it suggests that family environment (e.g., learning) is unlikely to have a major role in the etiology of the disorder. Instead, future studies should focus on the impact of specific environmental factors that are not shared between siblings or twins. Discordant sibling and twin designs are particularly suited to move the field forward because they effectively adjust for shared genetic factors and unmeasured confounders [3]. Using such designs, researchers have recently confirmed a dose-response relationship between perinatal complications and risk of OCD in the offspring [74].

Some limitations of the present study should be highlighted, such as the fact that the data were not analyzed according to the gender of the probands or the FDRs, to specific OCS subtypes or dimensions, to the symptom severity or the treatment response rates. These analyses could not be performed due to insufficient detail in many of the studies. For example, it seems likely that the tic-related subtype of OCD is particularly familial and heritable but limited data exists [75]. In addition, it would have been important to have more studies describing the recurrence risks for OCD according to the age of onset of OCS. Furthermore, Twin studies were based on self-reported questionnaires rather than on direct interviewed individuals but their results were largely compatible with those of the controlled family and population-based studies.

Despite these limitations, the current systematic review and meta-analysis represent a much needed update on the genetic epidemiology of OCD. The familial and heritable nature of OCD is now indisputable. In addition to large-scale gene-searching efforts, more needs to be done to understand environmental risk factors that are potentially modifiable, and how this newly gained knowledge can be used to improve the health of individuals with OCD and their relatives.

## Supplementary information

Box S1

Table S1

Table S2

Prisma 2020 Checklist
