# Supplementary material for: The genetic epidemiology of obsessive-compulsive disorder: a systematic review and meta-analysis
Source: Transl Psychiatry. 2023 Jun 28;13:230. doi: 10.1038/s41398-023-02433-2 (PMC10307810; doi:10.1038/s41398-023-02433-2)
Supplement: Supplementary file 1 — Box S1 [file 41398_2023_2433_MOESM1_ESM.docx]

**Box S1-** Detailed search strategy for each database

*- MEDLINE was searched using the following interface:*

((("Obsessive-Compulsive Disorder"[Mesh] or Disorder, Obsessive-Compulsive or Disorders, Obsessive-Compulsive or Obsessive Compulsive Disorder or Obsessive-Compulsive Disorders or Neurosis, Obsessive-Compulsive or Neuroses, Obsessive-Compulsive or Neurosis, Obsessive Compulsive or Obsessive-Compulsive Neuroses or Obsessive- Compulsive Neurosis or Anankastic Personality or Anankastic Personalities or Personalities, Anankastic or Personality, Anankastic))) AND (("Heredity"[Mesh] or "Genetic Predisposition to Disease"[Mesh] or Genetic Susceptibility or Genetic Susceptibilities or Susceptibilities, Genetic or Susceptibility, Genetic or Genetic Predisposition or Genetic Predispositions or Predispositions, Genetic or Predisposition, Genetic or heritability or Family study or twin study))

*- CENTRAL/Cochrane Library was searched using the following interface:*

(("Obsessive-Compulsive Disorder"[Mesh]) or (Disorder, Obsessive-Compulsive or Disorders, Obsessive-Compulsive or Obsessive Compulsive Disorder or Obsessive-Compulsive Disorders or Neurosis, Obsessive-Compulsive or Neuroses, Obsessive-Compulsive or Neurosis, Obsessive Compulsive or Obsessive-Compulsive Neuroses or Obsessive-Compulsive Neurosis or Anankastic Personality or Anankastic Personalities or Personalities, Anankastic or Personality, Anankastic)) and ((“heredity”[Mesh]) or ((Genetic Susceptibility or Genetic Susceptibilities or Susceptibilities, Genetic or Susceptibility, Genetic or Genetic Predisposition or Genetic Predispositions or Predispositions, Genetic or Predisposition, Genetic ) or (“Genetic Predisposition to Disease”[Mesh])) or (heritability or family study or twin study))

*- EMBASE was searched using the following interface:*

 ('ocd (obsessive compulsive disorder)'/exp OR 'ocd (obsessive compulsive disorder)' OR 'compulsion neurosis'/exp OR 'compulsion neurosis' OR 'compulsive neurosis'/exp OR 'compulsive neurosis' OR 'neurosis, compulsion'/exp OR 'neurosis, compulsion' OR 'neurosis, obsessive'/exp OR 'neurosis, obsessive' OR 'obsessional compulsive reaction'/exp OR 'obsessional compulsive reaction' OR 'obsessional neurosis'/exp OR 'obsessional neurosis' OR 'obsessive compulsive disorder'/exp OR 'obsessive compulsive disorder' OR 'obsessive compulsive neurosis'/exp OR 'obsessive compulsive neurosis' OR 'obsessive compulsive reaction'/exp OR 'obsessive compulsive reaction' OR 'obsessive neurosis'/exp OR 'obsessive neurosis' OR 'obsessive syndrome'/exp OR 'obsessive syndrome' OR 'obsessive-compulsive disorder'/exp OR 'obsessive-compulsive disorder' OR 'preoccupation neurosis'/exp OR 'preoccupation neurosis') AND ('genetic predisposition'/exp OR 'genetic predisposition' OR 'genetic predisposition to disease'/exp OR 'genetic predisposition to disease' OR 'genetic prognosis'/exp OR 'genetic prognosis' OR 'predisposition, genetic'/exp OR 'predisposition, genetic' OR 'genetic determinism'/exp OR 'genetic determinism' OR 'genetic effect'/exp OR 'genetic effect' OR 'genetic factor'/exp OR 'genetic factor' OR 'genetic phenomena'/exp OR 'genetic phenomena' OR 'genetic processes'/exp OR 'genetic processes' OR 'heredity'/exp OR 'heredity'OR 'familiy study' OR 'twin study'/exp OR 'twin study' OR 'heritability'/exp OR 'heritability') AND [embase]/lim

*- BVS was searched using the following interface:*

(tw:("Transtorno Obsessivo-Compulsivo" OR (neurose obsessiva compulsiva) OR (neurose obsessivo-compulsiva) OR (personalidade anancástica) OR mh:f03.080.600*)) AND (tw:("Hereditariedade" OR (herança genética) OR mh:g05.390* OR "Predisposição Genética para Doença" OR (predisposição genética) OR (suscetibilidade genética) OR mh:c23.550.291.687.500* OR mh:g05.380.355* OR estudos de família OR estudos de gêmeos OR herdabilidade)) AND (instance:"regional") AND ( db:("LILACS" OR "IBECS"))
