# Supplementary material for: The genetic epidemiology of obsessive-compulsive disorder: a systematic review and meta-analysis
Source: Transl Psychiatry. 2023 Jun 28;13:230. doi: 10.1038/s41398-023-02433-2 (PMC10307810; doi:10.1038/s41398-023-02433-2)
Supplement: Supplementary file 2 — Table S1 [file 41398_2023_2433_MOESM2_ESM.docx]

**Table S1**. Studies excluded from the meta-analysis.

| Study | Design | Reason for exclusion |
| --- | --- | --- |
| Clark et al., 1985 | Family study | Did not use standardized instruments for OCD diagnosis (self-report measure) |
| Rasmussen et al., 1986 | Family study | Did not use direct interview for OCD diagnosis in relatives (family history method) |
| Andrews et al., 1990 | Twin study | Reported polychoric correlations regarding only to neuroticism, not OCD. |
| Last et al., 1991 | Family study | Neither OCD was the main topic nor the probands were OCD-affected cases. |
| Schlosser et al, 1994 | Family study | Did not report frequency of OCD in relatives |
| King et al. 1995 | Family study | Did not report comparative family loading between cases and controls. |
| Sciuto et al., 1995 | Family study | Absence of non-OCD control group |
| Thomsen, 1995 | Family study | Did not describe the sample size of relatives of OCD probands and controls; did not use standardized instruments for OCD diagnosis. |
| Black et al., 1998 | Family study | Did not use direct interview for OCD diagnosis in relatives. |
| Albert et al., 2002 | Family study | Absence of control group |
| Rosario-Campos et al., 2014 | Family study | Sample e data report overlap with Rosario-Campos et al (2005). |
| Bhattacharyya et al. 2005 | Family study | Did not describe frequencies of OCD but OCS dimensions. |
| Chabane et al., 2005 | Family study | Absence of control group |
| Hanna et al., 2005 | Family study | Absence of control group; did not report frequency of OCD in relatives. |
| Samuels et al., 2006 | Family study | Study protocol report. |
| Hasler et al., 2007 | Family study | Absence of control group; did not report frequency of OCD in relatives. |
| Cullen et al., 2007  Cullen et al., 2008 | Family study | Did not describe frequencies of OCD in relatives. |
| Ettelt et al., 2007  Ettelt et al., 2008 | Family study | Did not describe frequencies of OCD in relatives. |
| Mathews et al., 2007 | Family study | Describe data from multigenerational families with OCD; absence of comparing group |
| van Grootheest et al., 2007 | Twin study | Compared mother and father ratings but not global rating. |
| de Braber et al., 2008 | Twin study | Did not report OCD heritability analysis and correlations, but association of neuroimaging with OC symptomatology in twin sample. |
| Cath et al., 2008 | Twin Study | Did not report OCD heritability analysis and correlations, but association of environmental factors with OC symptomatology in twin sample. |
| Mathews et al., 2008 | Family study | Did not use standardized instruments for OCD diagnosis (self-report measure) |
| Taberner et al., 2009  Taberner et al., 2012 | Family study | Did not use standardized instruments for OCD diagnosis. |
| Rector et al., 2009 | Family study | Did not describe frequencies of OCD in relatives. |
| Kateberg et al. 2010 | Family study | Data on OCD in relatives was assessed by positive family history for OC symptoms, not by direct interview; did not report non-OCD comparison group. |
| Taylor & Jang, 2010 | Twin study | Investigated correlations between dysfunctional beliefs and OCS. |
| Taylor et al., 2010  Taylor et al., 2010  Taylor et al., 2011 | Twin study | Investigate correlations between prototypic OCS and personality traits. |
| Mathews et al., 2011 | Family study | OCD was not the main topic, but Tourette syndrome. All proband sample comprised Tourette affected-cases. |
| Coskun et al., 2012 | Family study | Did not use standardized instruments for OCD diagnosis in relatives. |
| Monzani et al., 2012 | Twin study | Did not report data regarding to correlations of OCD occurrence among twins |
| Steinhausen et al., 2013 | Family study | Did not use standardized instruments for OCD diagnosis. Nationalwide register-based study (narrative synthesis) |
| Arumugham et al. 2014 | Family study | Diagnosis of OCD among family members was made by obtaining history from the index proband and a first-degree relative and not by direct interviews; did not describe frequencies of OCD and related disorders in relatives. |
| Bolhuis et al., 2014 | Twin study | Did not report data regarding to correlations of OCD occurrence among twins |
| Samuels et al., 2014 | Family study | Did not describe frequencies of OCD in relatives in cases and controls. |
| Alvarenga et al., 2015 | Family study | Did not use standardized instruments for OCD diagnosis. |
| Browne et al., 2015 | Family study | Did not use standardized instruments for OCD diagnosis. |
| Cederlof et al., 2015 | Family study | Did not use standardized instruments for OCD diagnosis; wrong outcome: did not describe frequencies of OCD in relatives. |
| Goldberg et al., 2015 | Family study | Absence of control group; did not describe frequency of OCD in relatives (just the familial loading coefficient). |
| Lopéz-Solà et al., 2015 | Twin study | Investigate correlations between OCS dimensions |
| Brander et al., 2016 | Family study | Did not investigate OCD familial loading but perinatal risk factor for OCD |
| Vidal-Ribas et al., 2015 | Twin study | Did not report data regarding to correlations of OCD occurrence among twins |
| Nissen et al., 2016 | Family study | Did not use standardized instruments for OCD diagnosis in relatives. |
| Ozcan et al., 2016 | Family study | Did not describe frequencies of OCD in relatives in cases and controls. |
| Sica et al., 2016 | Family study | Did not use standardized instruments for OCD diagnosis (self-report measure) |
| Petterson et al., 2018 | Family study | Did not use standardized instruments for OCD diagnosis. |
| Sidorchuk et al., 2019 | Family study | Did not use standardized instruments for OCD diagnosis. |
| Wu et al, 2019 | Family study | Did not describe frequencies of OCD in relatives. |

**papers unavailable in any resources for full text analysis*: Walkup JT, Leckman JF, Price RA, Hardin MT, Oft S, Cohen DJ (1988): The relationship between Tourette's syndrome and obsessive compulsive disorder: A twin study. Psychopharmacology Bull 24:375-379; Nicolini, H., Weissbecker, K., Mejía, J. M., & de Carmona Sánchez, M. (1993). Family study of obsessive-compulsive disorder in a Mexican population. Archives of medical research, 24(2), 193-198; Nicolini, H.; Orozco, B.; Mickalonis, L.; Mejia, J. M.; Paez, F.; Gomez, A.; De La Fuente, J. R (1998). A phenomenological and family study of obsessive compulsive disorder. Neuropsychiatrie de l'Enfance et de l'Adolescence, 46 (3), 164-172; Yang, Y; Liu, X (1998). Afamily study of obsessive-compulsive disorder]. Chinese journal of medical genetics, 15 (5), 303-6.; Nestadt, G., Samuels, J. F., Riddle, M. A., Bienvenu, O. J., Liang, K. Y., Grados, M. A., & Cullen, B. (2002). Obsessive-compulsive disorder: defining the phenotype. The Journal of clinical psychiatry, 63, 5-7.; Gonzalez, C. H. (2003). Estudo de familias no transtorno obsessivo-compulsivo.; Delorme, R.; Chabane, N. (2007) Genetics of obsessive compulsive disorder. Revue du Praticien, 57 (1), 48.; Taner, Y.; Taner, E.; Bakar, E. E.; Bodur, S. (2007). Psychopathology in first-degree relatives of pediatric obsessive-compulsive disorder patents. Anadolu Psikiyatri Dergisi, 8 (2), 126-131; den Braber, A., van't Ent, D., Blokland, G. A., van Grootheest, D. S., Cath, D. C., Veltman, D. J., ... & Boomsma, D. I. (2008). An fMRI study in monozygotic twins discordant for obsessive–compulsive symptoms. Biological psychology, 79(1), 91-102.; Browne, H. A.; Hansen, S. N.; Buxbaum, J. D.; Gair, S. L.; Nissen, J. B.; Nikolajsen, K. H.; Schendel, D. E.; Reichenberg, A.; Parner, E. T.; Grice, D. E. (2014) High familial clustering of tic disorders and OCD in a population-based cohort. Neuropsychopharmacology, 39 (0), S528-S529; López-Solà, C.; Fontenelle, L. F.; Menchón, J. M.; Alonso, P.; Harrison, B. J. (2014) Multivariate twin study of obsessive-compulsive spectrum & anxiety symptoms. Biological Psychiatry, 75 (9), 215S; Malhotra, S. (2016) A study of neurological soft signs in patients of OCD and their first-degree relatives: State marker or trait marker? Indian Journal of Psychiatry, 58 (5), S73-S74.; Zilhao, N. (2017) Cross disorder genetic analysis of Tourette's syndrome, obsessive compulsive disorder and hoarding. European Neuropsychopharmacology, 27 (0), S303.
