# Supplementary material for: The genetic epidemiology of obsessive-compulsive disorder: a systematic review and meta-analysis
Source: Transl Psychiatry. 2023 Jun 28;13:230. doi: 10.1038/s41398-023-02433-2 (PMC10307810; doi:10.1038/s41398-023-02433-2)
Supplement: Supplementary file 3 — Table S2 [file 41398_2023_2433_MOESM3_ESM.docx]

# Table S2 – Quality assessment criteria of the family studies according to a modified version of the Newcastle-Ottawa Scale for observational studies

|  | McKeon & Murray, 1987^38^ | Black et al., 1992^39^;  Black et al., 2013^4^ | Pauls et al., 1995^16^;  Carter et al. 2004^29^ | Bienvenu et al. 2000^40^;  Nestadt et al. 2000^18^;  Grados et al. 2001^23^;  Nestadt et al. 2001^24^ | Reddy et al., 2001^20^ | | Black et al., 2003^25^ | | Hanna et al., 2005^21^;  Hanna et al., 2011^22^ | | Rosario-Campos et al., 2005^17^ | Fyer et al., 2005^26^ | Lipsitz et al., 2005^27^ | Grabe et al., 2006^28^ | Bienvenu et al., 2012^5^ | Chabane et al., 2005^42^ | Sciuto et al., 1995^41^ | Bellodi et al., 1992^13^ |
| --- | --- | --- | --- | --- | --- | --- | --- | --- | --- | --- | --- | --- | --- | --- | --- | --- | --- | --- |
| Selection |  |  |  |  |  | |  | |  | |  |  |  |  |  |  |  |  |
| Case definition: a) using best estimate method * b) did not use best estimate method c) no description | c | c | * | * | * | | * | | * | | * | * | * | * | * | b | b | b |
| Representativeness of the cases: a) subjects diagnosed with OCD based on DSM or ICD versions * b) not stated | b  Research Diagnostic Criteria (RDC) | *  DSM-III | *  DSM-III-R | *  DSM-IV | *  DSM-III-R | | *  DSM-IV | | *  DSM-III-R | | *  DSM-IV | *  DSM-III-R | *  DSM-IV | *  DSM-IV | *  DSM-IV | *  DSM-IV | *  DSM-III-R | *  DSM-III-R |
| Controls selection: a) controls matched by age and gender * b) not matched procedure applied c) no description | * | * | c | * | * | | * | | b | | * | * | * | * | * | c | c | c |
| Definition of controls: a) mentally health controls or adequately acces of age and comorbidity status (when applied)* b) not stated | * | * | * | * | * | | * | | * | | * | * | * | * | * | * | * | * |
| Control for confounders: a) study reports assessment of risk for OCD or related disorders in relatives adjusted for age* b) study reports data of controlling statistical procedures for age and any additional factors** c) no description related to the adjustment analysis for confounding factor | ** | ** | * | ** | * | | ** | | ** | | ** | ** | ** | ** | ** | * | * | * |
| Assessment of relatives | |  |  |  |  |  | |  | |  |  |  |  |  |  |  |  |  |
| Ascertainment of relatives: a) interviewer blind to relative/proband status * b) interviewer not blind to relative/proband status c) no description | b | * | * | * | b | | * | | * | | * | * | * | * | * | c | c | c |
| Method of ascertainment: a) same for relatives of cases and controls or different instruments if required considering the age of subject under assessment * b) different assessment procedures or instruments between the same-age subjects c) no description | * | * | * | * | * | | * | | * | | * | * | * | * | * | * | * | * |
| Number (%) of relatives directly assessed: a) no significant difference between proportion of relative of case and control probands * b) statistical difference between the groups c) not stated | * | * | c | * | b | | c | | * | | * | * | * | * | b | * | * | * |
| SUMMARY SCORE | 6/9 (moderate) | 8/9  (high) | 7/9 (moderate) | 9/9  (high) | 6/9 (moderate) | | 8/9  (high) | | 8/9  (high) | | 9/9  (high) | 9/9  (high) | 9/9  (high) | 9/9  (high) | 8/9  (high) | 5/9  (low) | 5/9  (low) | 5/9  (low) |
